# Inequalities in health status among rural residents: EQ-5D findings from household survey China

**DOI:** 10.1186/1475-9276-13-41

**Published:** 2014-05-19

**Authors:** Haitao Li, Xiaolin Wei, Aixia Ma, Roger Y Chung

**Affiliations:** 1 School of Public Health and Primary Care, Faculty of Medicine, The Chinese University of Hong Kong, Hong Kong, China; 2 School of International Pharmaceutical Business, China Pharmaceutical University, Nanjing, China

**Keywords:** Inequality, EQ-5D, NRCMS, Rural, Health status

## Abstract

**Introduction:**

This study analyzed inequalities in health status among different socioeconomic and demographic rural residents covered by the New Rural Cooperative Medical System in China.

**Methods:**

A cross-sectional study was conducted in Lian Yungang City, China. A total of 337 respondents, who were selected by using a multistage stratified systematic random sampling method, completed the surveys. A questionnaire consisting of EQ-5D and demographic and socioeconomic information was adopted for data collection, and was administered by face-to-face interviews. Multiple regression models were employed to examine the differences in the Visual Analogue Scale (VAS) score and the EQ-5D dimensions.

**Results:**

Compared with those with lower education attainment, the respondents with higher education levels tended to report a higher VAS score (β = 2.666, 95% CI: 0.978 to 6.310), and were less likely to suffer from pain/discomfort (OR = 3.968; 95% CI: 1.447 to 10.880). The singles were more likely than the married to report moderate or extreme problems in usual activities (OR = 4.583; 95% CI: 1.188 to 17.676) and mobility (OR = 10.666; 95% CI: 2.464 to 6.171). However, no statistically significant differences were identified between the respondents with different income levels in the VAS score and EQ-5D dimensions.

**Conclusions:**

This study suggests that the singles and the people with lower education levels are high-risk groups for poorer health status in the Chinese rural population. The findings from this study warrant further investigation.

## Introduction

The pilot program of the New Rural Cooperative Medical System (NRCMS) was launched by the Chinese Government in 2003. Its main objectives were to promote access to health services and improve rural people’s health, and it is by far the largest state subsidized and linked micro-insurance system in the world. By the end of 2010, the coverage rate was greater than 97% and was considered to be almost universal among rural residents [1]. Since the implementation of the NRCMS, the general health of rural residents has improved greatly [1]. Yet we are not only concerned about the improvement of overall health of rural residents, but also the distribution of health among those with different socioeconomic and geographical characteristics. Reducing inequalities has been widely recognized as a major objective of health care policies in China and has become a growing concern among the public. Previous studies had evaluated the impact of the NRCMS on inequalities in health to test whether the NRCMS was leading toward or departing from greater social justice [2,3]. Those studies showed that the NRCMS tended to promote equitable distribution of health comparing those rural residents being covered by insurance with those not being covered. However, inequalities in health existed within rural residents covered by the NRCMS, and health was poorer among the less advantaged social groups than the more advantaged [4,5].

While these studies have provided valuable insights into the impact of the NRCMS on reducing inequality in health between different social groups in rural China, there are several limitations to these studies. The main deficiency is that health status measures adopted in these studies were either two-week morbidity rate or prevalence rate of chronic diseases. Along with the development of the new medical model (bio-psychosocial), the demographic transition with increasing number of elderly and the epidemiologic transition with chronic diseases as the predominant leading causes of morbidity and mortality, traditional measurements of health such as incidence rate or prevalence rate become insufficient. Over the past decade, there has been a growing acceptance of the need for a more systematic record on health status.

EuroQol (EQ-5D) is a standardized instrument to systematically measure and record health status for individuals or a population. EQ-5D instrument has widely been used in North America, Europe and Asia (including China) [6-11]. A study using the Chinese sample in Beijing indicated that EQ-5D was valid for measuring health status in the Chinese population [12]. Two studies performed by Sun et al. [13,14] in 2008 used EQ-5D to measure the Chinese population’s health status and to estimate regional differences. It is also worth noting the two studies about inequality in health status among rural residents in China used EQ-5D measures. Zhang et al. [15] analyzed the health-related quality of life (HRQOL) of 2,830 rural residents using EQ-5D, and showed that HRQOL of rural population was relatively low and there were statistically significant differences between advantaged and disadvantaged groups. However, the study was conducted in 2002, one year before the implementation of the NRCMS. On the other hand, the other health inequality study that used EQ-5D did not give definite information regarding whether the recruited rural residents were covered by the NRCMS or not [14].

The aim of the present study is to describe and analyze potential inequalities in health status among residents with different demographic and socio-economic groups covered by the NRCMS in rural China. EQ-5D measures will be employed to fill the gap in the literature.

## Methods

### Study area and sample design

Lian Yungang City is situated in the northeastern part of the Jiangsu Province, China, administering seven county-level divisions, including three districts and four counties. By the end of 2010, the number of rural residents was approximated at 3.3 million, accounting for about 75% of the total population in Lian Yungang. In the same year, more than 99% of rural residents were registered with the NRCMS [16].

**Figure 1:**
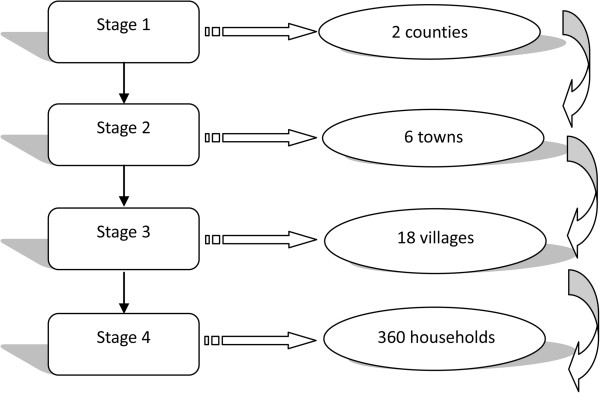
**lllustates the sampling process of the present study.** The sampling involved four stages. In the first stage, with counties as the sampling frame, we randomly selected two counties from Lian Yungang City. Then, with towns within the two counties as the sampling frame, we chose six towns for the two counties at random. Afterwards, using the same sampling method as in the second stage, a total of 18 villages were chosen. At last, we used systematic random sampling methods to select 360 households.

Employing a complex multistage stratified random sampling method, a total of 360 respondents were planned to be interviewed. The target population consisted of all rural residents aged 18 and above who lived in households of Lian Yungang City. The sampling involved four stages. In the first stage, we used all four counties in Lian Yungang City as the sampling frame. The four counties were then divided into two strata according to the average income of rural residents of each county. We then randomly selected one county from each stratum and two counties in total were chosen. In the second stage, with towns within the two counties as the sampling frame and income as strata, we divided the towns of each county into three groups. We then randomly selected one town from each group. Altogether, six towns were chosen for the two counties. In the third stage, we employed the same sampling method as in the second stage, but with villages as the sampling frame. A total of 18 villages were chosen. In all three stages, simple random sampling method was used. In the last stage, we used systematic random sampling methods to select households on the basis of household roster. Twenty households were selected from each village, giving 360 households in total. Please refer to Figure 1 for an illustration of the sampling process of the study. Inclusion criteria of the respondents were 1) age of 18 years or older, 2) ability to communicate and give informed consent, and 3) last birthday closest to the date of the interview (to minimize over-representation of housewives and the elderly). In the end, 337 respondents completed the survey with a response rate of 94%.

### Survey procedure

The household survey was conducted between July and August of 2010. Face-to-face interviews were performed by trained interviewers from China Pharmaceutical University. A pretested standard questionnaire was used to collect information from the respondents. Supervisors were responsible for the quality control during the whole process of the data collection, including the completeness of the questionnaire and the logical rationality of the reported information. Before the survey, the respondents were informed the objectives of the study, and were assured of the anonymity and confidentiality of the survey. Informed consent was obtained before the surveys commenced. Agreement was also reached between the researchers and the respondents on the use of the data for scientific purposes. The study was approved by the Ethical Committee of China Pharmaceutical University.

### Variables for socioeconomic and demographic categorizations

Demographic data such as gender, age, marital status, family size; and socioeconomic data including occupation, education and income were collected in the survey. Marital status was classified into two categories - “married” and “single.” “Single” included people who were never married and people who were currently divorced or widowed at the time of the survey administration. “Married” people were currently married. Rural residents with low level of education were those who attained education of elementary school or below (schooling age ≤ 6 years). Others who had more than 6 years of schooling were classified into the high level of education attainment group. For occupational status, we compared farmers with other people in rural areas, including students, self-employed, peasant-workers and the retired. According to the National Bureau of Statistics of China [17], peasant worker referred to individual who possessed an agricultural residence registration but did not engage in the agriculture work for more than 6 months during the past year, while farmers were defined as the population who possessed an agricultural residence registration and engaged in the agriculture work for more than 6 months in the past year. We also collected data on annually household income and number of household members. We classified the respondents into two income groups, i.e., those with low income and those with high income, based on the annually per capita income (i.e., 5,000 RMB/US$643) of rural residents in Lian Yungang City in 2010 [16].

### EQ-5D instrument

The EQ-5D was developed by the EuroQol Group, a voluntary multinational collaboration of European investigators [18]. The EQ-5D had been translated into Chinese, and shown substantial validity and reliability in various studies [12,19,20]. The EQ-5D defined health in five dimensions, including mobility, self-care, usual activities, pain/discomfort and anxiety/depression. The responses to each dimension were classified into three levels of severity - “1, no problem,” “2, some problem” and “3, extreme problem.” Health status in each dimension was presented as the percentage of the respondents reporting any problem in the corresponding dimension. We converted the three levels of responses into two categories - 1) any problem (including level “2, some problem” and “level 3, extreme problem”), and 2) no problem, since studies have shown that the EQ-5D instrument had ceiling effects in measuring health status of general population [21]. In addition to the five dimensions, a Visual Analog Scale (VAS) was incorporated for self-rating of overall health status, which was a 20 cm health thermometer with two end points being labeled as the “worst imaginable health state” and the “best imaginable health state”, ranging from 0 to 100. The respondents were asked to mark a point on the scale to indicate how good or bad his/her own overall health status was, with a higher score indicating a better self-reported overall health status [18].

### Statistical analysis

In this study, SPSS version 18.0 (IBM Corporation) was employed to analyze the data. The percentages of respondents reporting any problem in each EQ-5D dimension were calculated. The Chi-square test and multiple logistic regression analysis were used to examine whether or not significant differences in each of the EQ-5D dimensions existed between groups. The VAS score was analyzed in accordance with different socioeconomic and demographic characteristics employing independent two samples *t*-test (or ANOVA where appropriate) and multiple linear regression analysis. For all tests conducted in the study, a p-value of 0.05 or below was adopted as the statistically significant level.

## Results

### Demographic and socioeconomic characteristics of respondents

Of the 337 respondents, 63.5% were male and 36.5% were female. More than half of the respondents (51.3%) were 18 to 44 year-old, 41.8% of the respondents aged from 45 to 64 year-old, and the rest (6.8%) were 65 years or older. 84.6% of the respondents were married, and 15.4% were single. 28.2% of the respondents had a low level of education, while 71.8% had a high level of education. The respondents were almost equally distributed between the two income groups - 52.2% and 47.8% for low- and high- income groups respectively. Farmers accounted for 55.2% of the total respondents, and the rest (44.8%) were students, peasant-workers, self-employed and the retired (Table 1).

**Table 1:** Demographic and socioeconomic characteristics of the respondents

**Indicators**	**Number (%)**
Number of respondents	337 (100.0)
Gender	
Male	214 (63.5)
Female	123 (36.5)
Age group	
18-44	173 (51.3)
45-64	141 (41.8)
65+	23 (6.8)
Marital status	
Single (divorced or widowed)	52 (15.4)
Married	285 (84.6)
Education	
Low level	95 (28.2)
High level	242 (71.8)
Income group	
Low level	176 (52.2)
High level	161 (47.8)
Occupation	
Farmer	186 (55.2)
Others	151 (44.8)

### Differences of the five dimensions by socio-economic and demographic status

Table 2 shows the percentages of respondents reporting any problem in each EQ-5D dimension. When compared with those younger counterparts, the older respondents were more likely to report any problem in every EQ-5D dimension with statistical significance (*p* < 0.05), except for anxiety/depression. The singles were more likely than the married to suffer from anxiety/depression (*p* =0.037), and to constrain themselves to usual activities (*p* =0.048) and to restrict mobility (*p* = 0.003). Compared with the better educated, the less educated were more likely to suffer from pain/discomfort (*p* = 0.002), and to restrict their mobility (*p* = 0.048). The percentages of reporting any problem in the anxiety/depression (*p* = 0.036) and pain/discomfort (*p* = 0.043) dimensions were higher among farmers than other rural residents including students, peasant-workers, self-employed and the retired.

**Table 2:** Percentage of respondents reporting moderate or severe problems in each EQ-5D dimensions

**Indicators**	**Usual activities**		**Anxiety/depression**		**Pain/discomfort**		**Mobility**		**Self-care**	
	**% of any problems**	**P-value**	**% of any problems**	**P-value**	**% of any problems**	**P-value**	**% of any problems**	**P-value**	**% of any problems**	**P-value**
Gender										
Male	8(3.7)	0.586	15(7.0)	0.829	15(7.0)	1.000	7(3.3)	1.000	4(1.9)	0.709
Female	6(4.9)		10(8.1)		9(7.3)		4(3.3)		3(2.4)	
Age group										
18-44	4(2.3)	0.003**	13(7.5)	0.527	6(3.5)	0.012*	4(2.3)	<0.001***	1(0.6)	<0.001***
45-64	6(4.3)		9(6.4)		14(9.9)		3(2.1)		3(2.1)	
65+	4(17.4)		3(13.0)		4(17.4)		4(17.4)		3(13.0)	
Marital										
Single	5(9.6)	0.048*	8(15.4)	0.037*	4(7.7)	0.774	6(11.5)	0.003**	2(3.8)	0.295
Married	9(3.2)		17(6.0)		20(7.0)		5(1.8)		5(1.8)	
Education										
Low level	7(7.4)	0.064	10(10.5)	0.174	14(14.7)	0.002**	6(6.3)	0.048*	3(3.2)	0.407
High level	7(2.9)		15(6.2)		10(4.1)		5(2.1)		4(1.7)	
Income group										
Low level	9(4.6)	0.603	14(7.2)	0.869	11(5.7)	0.228	7(3.6)	0.765	6(3.1)	0.246
High level	5(3.5)		11(7.7)		13(9.1)		4(2.8)		1(0.7)	
Occupation										
Farmer	10(5.4)	0.277	19(10.2)	0.036*	18(9.7)	0.043*	6(3.2)	1.000	4(2.2)	1.000
Others	4(2.6)		6(4.0)		6(4.0)		5(3.3)		3(2.0)	

Note: P-value: chi-square test; *P < 0.05; **P < 0.01;***P < 0.001; Bolded: statistically significant.

Using multiple logistic regression models, the likelihood of having any problem in each EQ-5D dimension was analyzed for all socioeconomic and demographic characteristics after controlling for other influencing factors. The statistically significant difference in pain/discomfort dimension between each age group disappeared. Although the singles were more likely to report any problem in anxiety/depression dimension, the difference was not statistically significant anymore. Similar pattern was found in mobility dimension between groups with different education levels. There were no statistically significant differences anymore between the farmers and other rural residents (including students, peasant-workers, self-employed and the retired) in both anxiety/depression and pain/discomfort dimensions (Table 3).

**Table 3:** Multiple logistic regression analysis on having any problems in EQ-5D dimensions

**Indicators**	**Usual activities**		**Anxiety/depression**		**Pain/discomfort**		**Mobility**		**Self-care**	
	**Odds ratio**	**95% CI**	**Odds ratio**	**95% CI**	**Odds ratio**	**95% CI**	**Odds ratio**	**95% CI**	**Odds ratio**	**95% CI**
Gender										
Male	1.000		1.000		1.000		1.000		1.000	
Female	1.486	(0.464,4.766)	1.104	(0.464,2.626)	1.060	(0.425,2.643)	1.202	(0.297,4.859)	1.833	(0.349,9.633)
Age group										
18-44	1.000		1.000		1.000		1.000		1.000	
45-64	2.964	(0.636,13.807)	0.974	(0.347,2.732)	2.454	(0.807,7.460)	1.132	(0.182,7.029)	9.149	(0.724,115.670)
65+	**7.724**	**(1.380,43.238)**	1.227	(0.276,5.447)	3.843	(0.868,17.010)	**7.575**	**(1.191,48.179)**	**47.390**	**(3.179,706.385)**
Marital										
Married	1.000		1.000		1.000		1.000		1.000	
Single	**4.583**	**(1.188,17.676)**	2.607	(0.956,7.112)	1.210	(0.348,4.207)	**10.666**	**(2.464,46.171)**	5.403	(0.692,42.202)
Education										
High level	1.000		1.000		1.000		1.000		1.000	
Low level	1.412	(0.358,5.566)	1.847	(0.669,5.094)	**3.968**	**(1.447,10.880)**	2.676	(0.496,14.437)	0.308	(0.043,2.219)
Income group										
High level	1.000		1.000		1.000		1.000		1.000	
Low level	1.387	(0.354,5.435)	1.103	(0.427,2.851)	0.387	(0.142,1.056)	0.896	(0.170,4.718)	7.181	(0.591,87.212)
Occupation										
Farmer	1.000		1.000		1.000		1.000		1.000	
Others	0.574	(0.161,2.050)	0.426	(0.157,1.156)	0.413	(0.151,1.132)	2.190	(0.494,9.702)	1.001	(0.184,5.460)

Note: CI: Confidence interval; Bolded: statistically significant.

### Differences of VAS score by socio-economic and demographic status

The younger respondents tended to report a higher VAS score than their older counterparts (β = −0.217, 95% CI: −0.342 to −0.092). Compared with those in the lower education group, the respondents in the higher education attainment group tended to report higher VAS scores (β = 2.666, 95%CI: 0.978 to 6.310). Statistically significant differences were not found in VAS score among other socio-economic and demographic groups (Table 4).

**Table 4:** VAS score reported by respondents and multiple linear regression analysis

**Indicators**	**Single factor analysis**			**Multiple linear analysis**	
	**Mean**	**SD**	**P-value**	**β-estimate**	**95% CI**
Gender					
Male	82.379	12.029	0.967	0.000	
Female	82.317	15.019		−0.418	(−3.331,2.496)
Age group				**−0.217**	**(−0.342,-0.092)**
18-44	85.740	10.518	<0.001***		
45-64	79.135	14.526			
65+	76.652	15.925			
Marital					
Single	83.596	15.265	0.461	0.000	
Married	82.130	12.777		2.401	(−1.871,6.672)
Education					
Low level	78.374	15.776	<0.001***	0.000	
High level	83.930	11.670		**2.666**	**(0.978,6.310)**
Income group					
Low level	81.949	13.296	0.509	0.000	
High level	82.949	13.039		−0.651	(−3.636,2.333)
Occupation					
Farmer	81.397	13.573	0.229	0.000	
Others	83.134	12.831		1.852	(−1.039,4.742)

Note: P-value: independent two-sample *t*-test or one-way ANOVA; ***P < 0.001; Bolded: statistically significant; CI: confidence interval; VAS: visual analog scale; SD: Standard deviation.

## Discussion

This is the first study that used EQ-5D measures, instead of the commonly used mortality and morbidity indicators, to investigate the inequalities in health status among rural residents with different socioeconomic and demographic groups who were covered by the NRCMS. It was found that the respondents with higher education levels tended to report a higher VAS score and were less likely to suffer from pain/discomfort when compared to those with lower education levels; and the results stayed unchanged with or without adjustments. Compared to the married, the singles were more inclined to have any problem in usual activities and mobility. No statistically significant differences were identified in VAS score and EQ-5D dimensions among the respondents of different income groups.

The limitations of the study should be addressed. Firstly, there might be response bias although the response rate was high. People in low socioeconomic groups are less likely to answer the survey, but it is difficult to assess the potential bias introduced by this limitation. Secondly, the high percentage of male respondents might introduce the selection bias. Thirdly, there might be information bias. All information regarding health status is self-reported and thus our estimates might be subject to the respondents’ mental status at the time of the surveys commenced which could not be accounted for by statistical adjustments. Lastly, the generalizability of the study results to other regions is limited. The present study took place in Lian Yungang City and the results are applicable to its specific context. In other words, regional policy makers should formulate contextually specific policies according to their own regionally specific survey results.

The respondents of higher education level tended to report higher VAS scores, and were less likely to report any problem with respect to pain/discomfort, when compared with those with lower education attainment. Our findings were in agreement with those observed in previous studies. The study by Zhang and colleagues [15], which was conducted in rural West China, showed that more education was predictive of higher VAS scores. The study conducted by Sun et al. [13] in 2008 using a nationally representative sample also indicated that individuals with higher education levels tended to report higher mean VAS scores. Moreover, the study by Zhang et al. [15] demonstrated that lower education attainment was associated with higher probability of reporting any problem in pain/discomfort dimension. Studies suggested that education can influence health status directly or through its vehicle mechanisms such as reduced workload [22]. People with higher education levels usually have better knowledge of health, and tend to engage in more health promoting activities (e.g., physical exercise). Moreover, with better knowledge of the health care system, they can communicate with health providers more effectively, and have better access and utilization of the health care services [23-26]. Furthermore, the better-educated individuals show better self-management and compliance, which translate into better treatment results [27]. The advantage of a high education level may weigh even more in rural China, where the NRCMS was implemented without sufficient amount of campaigns to educate the general public when it was first introduced [28]. The rural residents with higher education level might thus have more cognition and knowledge of the NRCMS insurance scheme, which may possibly translate into more utilization of health services [29,30].

On the other hand, the singles were found to be more likely to report any problem in usual activities and mobility dimensions which may reflect the supportive role of the marital partner. It is commonly reported in the literature that the married tend to live longer and are healthier than the singles [31,32]. It has been hypothesized that marriage increases social support and income, and also reduces risky behaviors [33]. However, in our study, we did not identify any significant difference in overall self-reported health status between the singles and the married. This warrants further investigations.

No notable differences were found in the current study regarding variations in the reported VAS scores and EQ-5D dimensions between the respondents with different income levels. Previous studies showed that people with lower income levels often were less healthy than those with higher income levels [34,35]. Both studies by Zhang et al. [15] and Sun et al. [13] indicated that people with lower income levels were more likely than those with higher income levels to report any problem in EQ-5D dimensions such as mobility and pain/discomfort, although there was no statistically significant difference in VAS scores. The conflicting findings of the present study with the previous ones are possibly due to the fact that people in lower income groups are less likely to recognize and report any health problem due to their lower expectation of their health than individuals in higher income groups, given the same health condition [36]. In addition, in our study, the rural residents with higher income tended to be self-employed or peasant workers (data not shown). The self-employed respondents are usually fishermen who bear high workload and are more likely to be harmed, while the peasant workers in cities or towns are generally less skilled and minimally educated, and thus tend to engage in manual work that may also incur greater chances to be harmed. Therefore, health inequalities across the income strata may be underestimated, and statistically significant difference cannot be observed.

Health, like education, is among the basic capabilities that gives value to human life [37]. It contributes to both social and economic prosperity. However, the biases and discrimination that lead to differences in opportunities for health among social groups oppose the ideology that health is a basic human right, where everyone has the right to enjoy the highest attainable standard of health in their society [38]. It is therefore important for a society to protect its citizens’ health. From the findings of the present study, the government should allocate more resources to educate rural populations especially those of lower education level to improve their health knowledge (e.g., healthy lifestyle), including education on the NRCMS. Social support may also be provided to the singles to deal with the problems of usual activities and mobility. Even though peasant workers and fishermen have higher income than their rural counterparts, their workload is still high, and special attention should be paid to these high-risk groups. In this study, age is an important predictor of experiencing problems on the usual activities, mobility and self-care, and the overall self-reported health status. But due to the small sample size, we cannot compare the results among respondents of different socioeconomic groups by age. Further studies with larger sample size are therefore needed to assess socioeconomic and demographic inequalities.

## Conclusion

In conclusion, our study demonstrated that inequalities existed in different socioeconomic and demographic rural groups covered by the NRCMS. Future policies should target the singles and the people with lower education level, because they tend to belong to the high-risk groups for poorer health status in the Chinese rural population. The findings from this study warrant further investigation with larger sample size on the influence of income on health status.

## Competing interests

The authors declare that they have no competing interests.

## Authors’ contributions

XLW, HTL and RC conceived the study, and took part in its design. HTL and AXM participated in the data collection and data analysis. HTL and RC drafted the manuscript and were responsible for data interpretation. All authors read and approved the final manuscript.
